# Teaching provision for old age psychiatry in medical schools in the UK and Ireland: a survey

**DOI:** 10.1192/pb.bp.116.055210

**Published:** 2017-10

**Authors:** Sophia Bennett, Poppy Ilderton, John T. O'Brien, John-Paul Taylor, Andrew Teodorczuk

**Affiliations:** 1 Newcastle University, Newcastle, UK; 2 University of Cambridge, Cambridge, UK; 3 Griffith University, Queensland, Australia

## Abstract

**Aims and method** This work builds on a survey first done in 1999 to understand how old age psychiatry teaching is embedded in undergraduate medical schools in the UK and Ireland and the influence of academic old age psychiatrists on teaching processes. We invited deans of 31 medical schools in the UK and Ireland in 2015 to complete an online survey to reassess the situation 16 years later.

**Results** Response rate was 74%. As found in the original survey, there was variation across medical schools in how old age psychiatry is taught. Half of schools stated there was not enough space in the curriculum dedicated to old age psychiatry, and not all medical school curricula offered a clinical attachment. Medical schools that involved academic old age psychiatrists in teaching (59%) showed a greater diversity of teaching methods.

**Clinical implications** There is a need to recognise the importance of old age psychiatry teaching, with the consensus of opinion continuing to be that more curriculum space needs to be given to old age psychiatry. To achieve this we advocate increasing the number of old age psychiatrists with teaching roles, as relying on academics to teach and lead on curriculum development is challenging given their greater research pressures.

With the ageing population and high prevalence of mental health burden in the UK, it is becoming increasingly important that medical school undergraduate curricula for old age psychiatry advance in line with future demographic needs. This has been recognised by the General Medical Council (GMC) in *Tomorrow's Doctors*,^1^ which stresses the importance of students learning about the special problems associated with older people's health. The Royal College of Psychiatrists has subsequently mapped the core undergraduate psychiatry curriculum outcomes^2^ on to the competencies listed in *Tomorrow's Doctors.* Other UK national drivers, including the National Health Service (NHS) Outcomes Framework^3^ and the prime minister's dementia strategy,^4^ have highlighted the need for the future workforce to be competent in caring for the needs of the older person.

More broadly, there is also an increasing recognition that there is a shortage of old age psychiatrists, and recruitment of trainees is less than for other specialties (fill rate is 67% for training posts compared with 80% in psychiatry as a whole). The Centre for Workforce Intelligence (CfWI) in their in-depth review of the psychiatry workforce^5^ highlighted old age psychiatry as a particular concern, with the strongest risk of a larger demand–supply shortfall due to weak workforce growth. Currently, there are 1.1 full-time old age psychiatry certificate of completion of training (CCT) post holders per 100 000 population, but the workforce growth is not proportional to the growth in the older population, and baseline demand and supply projections anticipate a shortage of around 315 CCT holders in old age psychiatry by 2033.

In 2012, the Royal College of Psychiatrists published its recruitment strategy,^6^ with the primary aim of increasing recruitment into core psychiatry training, but the recommendations can be extended to old age psychiatry specialty training. Arguably, the concept of increased exposure to old age psychiatry and looking at the undergraduate experience could be key to understanding why medical school graduates may or may not consider this specialty as an appealing career choice.

This study builds on a previous survey, carried out in 1999, which found that schools with established old age psychiatry academic departments devoted more time to undergraduate teaching of old age psychiatry, covered more topics and used a wider range of teaching methods.^7^ The aim of the current study was to determine how old age psychiatry teaching is embedded in undergraduate medical schools in the UK and Ireland. More than 15 years on from the original survey, we sought to demonstrate how undergraduate provision of old age psychiatry teaching has changed nationally and identify any ongoing gaps. Similar surveys have been carried out by other non-psychiatric specialties facing similar recruitment concerns, in particular geriatric medicine.^8,9^

## Method

The study took place between January and November 2015. A questionnaire (available in the appendix) was developed and published in electronic format using the Survey Monkey software (www.surveymonkey.com). It enquired about the teaching provision of undergraduate old age psychiatry, covering whether or not the curriculum included teaching of old age psychiatry; where in the curriculum this teaching took place; whether academic old age psychiatrists (i.e. person(s) working in old age psychiatry but with a significant aspect of their job plan including research and/or medical education) were involved in the organisation of teaching; the duration of attachment to the specialty; the form and content of the teaching; and the nature of the student assessment. There were free-text spaces for respondents to qualify their answers if necessary. The questionnaire was reviewed and the study endorsed by the Faculty of Old Age Psychiatry of the Royal College of Psychiatrists.

The deans of all 31 UK medical schools were contacted by email and letter, asking them to identify a respondent with sufficient awareness of the undergraduate curriculum to allow completion of the survey, and requesting that the letter or email be forwarded to them. Where initial approaches and reminders were unsuccessful, members of the undergraduate leads forum were contacted through the Faculty of Old Age Psychiatry and invited to identify a respondent. As this was a survey requesting information already in the public domain, no formal consent procedure was undertaken and implied consent was assumed through participation.

The project met the Newcastle University preliminary ethical assessment guidelines, indicating that a full university ethics committee review was not required.

## Results

Responses were received from 23 (74%) medical schools. No schools responded that they did not wish to participate but 8 did not respond to any form of communication (written, electronic and/or telephone). One (4%) response was from a dean, 9 (40%) from a senior lecturer, 5 (22%) from a consultant psychiatrist or programme director, 3 (13%) from a reader in old age psychiatry, 4 (17%) from a professor and 1 (4%) from a consultant physician.

Overall, 50% of respondents felt that there was not sufficient curriculum space designated to old age psychiatry; however, many appreciated the overlap with other specialties, including adult psychiatry, neurology and care of the elderly.

### Staffing establishment of academic old age psychiatry

**Fig. 1:**
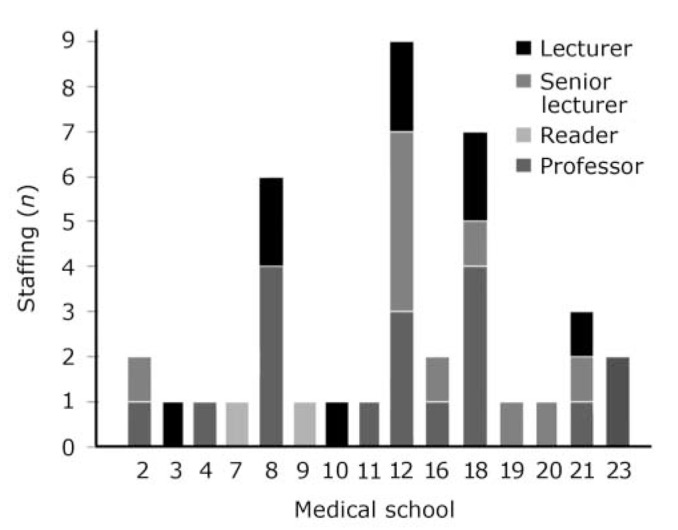
Staffing establishment of academic old age psychiatrists.

Figure 1 shows the current staffing establishment of academic old age psychiatry. More than half of schools (59%) reported that academic old age psychiatrists were involved in the organisation and delivery of undergraduate teaching, compared with 40% in the original survey. We found that 41% of schools have an academic old age psychiatrist represented on the board of studies or equivalent.

### Organisation and delivery of teaching

A designated clinical attachment in old age psychiatry was present in 86% of schools. Of those, in 13% the duration of the clinical attachment was dependent on individual student choice and in 41% there was the opportunity to spend more time in old age psychiatry. All respondents indicated that the majority of the teaching on cognitive assessment and dementia was delivered by an old age psychiatrist. There was no consistency between schools on when these topics were first covered, with 14% covering them in the first year, 23% in the second year, 36% in the third year, 23% in the fourth year and 5% in the fifth year.

With regard to time spent teaching old age psychiatry in the undergraduate curriculum, again there was no consistent standard, and reports ranged from a 1 h formal lecture to 25 days if the teaching on dementia/delirium covered by other specialties was included. This amount of time overlaps with that found in the original survey, in which time ranged from 1 to 40 days. Both the present and previous survey^7^ found that other, more informal/unstructured teaching was also given, but there was difficulty in quantifying the amount as this tends to depend on the clinical attachment. Some schools also offered self-directed teaching through podcasts or e-lectures (lectures delivered online), which depended on student interest and motivation.

A variety of teaching methods were used (Table 1), but with a large proportion primarily using lectures and tutorials as the main format of teaching (82% and 86%, respectively, *v*. 73% and 55% in the original survey). As with the original survey, schools where academic old age psychiatrists were involved in the organisation and delivery of teaching showed greater teaching method diversity and more use of innovative methods of teaching, such as interprofessional learning, e-learning and simulation, than those schools where there was no academic involvement. However, the overall figures were low (Table 1) and significance was only reached for simulation (*P* = 0.03) and home visits (*P* = 0.02). Of all schools, 50% responded that they involved carers or patients when delivering organised teaching sessions.

**Table 1:** Method of teaching delivery

	*n* (%)		
Teaching method	Schools with academic old age psychiatrist involvement (*N* = 13)	Schools without academic old age psychiatrist involvement (*N* = 10)	χ^2^ (*P*)
Lectures	12 (92)	6 (60)	0.13

Tutorials	11 (85)	8 (80)	0.57

Bedside teaching	10 (77)	7 (70)	1.00

Problem-based learning	5 (38)	4 (40)	1.00

Interprofessional education	7 (54)	3 (30)	0.40

e-learning	8 (62)	2 (20)	0.09

Simulation	5 (38)	0 (0)	0.03*

Home visits	13 (100)	6 (60)	0.02*

Joint teaching	6 (46)	2 (20)	0.38

Other	0 (0)	1 (10)	0.43

Binomial probability *P* = 0.03 for all the teaching methods where there is academic involvement.

* *P* ⩽ 0.05.

### Content and assessment of old age psychiatry teaching

In 95% of schools the undergraduate curriculum contained specific old age psychiatry outcomes, compared with 100% of schools in the original survey, where the undergraduate curriculum contained some theoretical or clinical teaching of old age psychiatry. All schools were teaching cognitive assessment and dementia (95% in the original survey) but, as found in the original survey, fewer indicated that they covered affective disorders (82% *v*. 91%) or psychotic disorders (77% *v*. 82%). Even fewer covered service organisation (36% *v*. 59%) (Table 2). A variety of assessment methods are used (Table 3), with the most common (82%) being the observed structured clinical examination (OSCE). In the original survey, student assessment was most commonly by ‘formal examination’ (68%) and assessment of performance during the clinical attachment (64%).

**Table 2:** Teaching content

Topic	Schools with academic old age psychiatrist involvement (*N* = 13)	Schools without academic old age psychiatrist involvement (*N* = 10)	χ^2^ (*P*)
Cognitive assessment	13 (100)	10 (100)	1.00

Dementia	13 (100)	10 (100)	1.00

Delirium	10 (77)	10 (100)	0.23

Affective disorders	10 (77)	8 (80)	1.00

Psychotic disorders	9 (69)	8 (80)	0.66

Service organisation	4 (31)	4 (40)	0.69

Mental Health Act	11 (85)	7 (78)	0.62

Cultural issues	7 (54)	4 (44)	0.68

Other	2 (15)	0 (0)	0.49

**Table 3:** Teaching and assessment methods in old age psychiatry

Assessment	Schools (*N* = 23) *n* (%)
Formal examination	11 (49)

OSCE	18 (82)

Long case	4 (18)

MCQ	14 (63)

Coursework	6 (27)

e-portfolio	3 (13)

Logbook	10 (45)

Essay	4 (18)

MCQ, multiple-choice questions; OSCE, observed structured clinical examination.

Table 2 also shows the content of teaching according to whether there is academic involvement which did not reach significance for any topic.

## Discussion

Arguably, the most powerful message from this survey is that 50% of respondents did not feel that sufficient curriculum space is designated to old age psychiatry. This had been a concern in the original survey, in which 57% of schools had reported that there were significant obstacles to introducing and maintaining old age psychiatry teaching in the undergraduate curriculum. This raises the question of how much progress has been made over the past 15 years. Similar surveys done in elderly care medicine, a specialty facing a similar recruitment problem, have also found that inadequate time (<2 weeks) is spent teaching about subjects related to ageing, including dementia, which does not reflect the predominance of older patients in most doctors' workload.^8,9,10^

The main finding in the original survey undertaken in 1999 had been that those medical schools with established academic old age psychiatry departments provided more teaching of old age psychiatry and are more likely to embrace new teaching methods.^7^ Our survey showed that 59% of schools have academic old age psychiatrists involved in the organisation and delivery of undergraduate teaching and 41% have an academic old age psychiatrist represented on the board of studies or equivalent. Similar to the original survey the main finding from the current survey is that schools where academic old age psychiatrists are involved in the organisation and delivery of teaching are more likely to use a greater diversity of teaching methods; however, only the use of simulation and home visits were found to be significant.

These results should be interpreted in the context that academics now may be less engaged in organising teaching (unless they are specifically medical educators) as they have predominantly research roles and greater research pressures. This highlights a need to drive up the number of other old age psychiatrists with teaching roles.

In the original survey all of the schools reported that the undergraduate curriculum contained some theoretical and/or clinical teaching of old age psychiatry and in our survey 95% of schools reported that their undergraduate curriculum contains specific old age psychiatry outcomes. Worryingly not all schools offer a clinical attachment in old age psychiatry (86%, slightly higher than in the original survey (82%)), and in those that did, the organisation of this is variable. As with the original survey, the amount of time offered varies considerably, and in some schools student exposure to old age psychiatry depends on individual clinical attachments. A wide range of teaching formats are reported, with the commonest methods being lectures and tutorials. Other methods, such as interprofessional teaching, e-learning and simulation, are less common (Table 1). Similarly, there was relatively low patient and carer involvement in teaching (50%).

Low use of interprofessional teaching and involvement of patients and carers in particular are missed opportunities, as evidence has shown that interprofessional education (IPE) can be used to significantly improve confidence and change attitudes in staff managing older patients with dementia or delirium.^11^ This style of teaching delivery could therefore also be applied to undergraduates, especially at a time when the expectation from the GMC is for greater IPE within curricula to improve team-working skills.^1,11^ As such, teaching on topics relevant to old age psychiatry could be the hook through which it is possible to drive up the amount of IPE, and hence development of team-working skills within the broader medical curricula, as well as fostering more positive attitudes towards the older patient and improving recruitment into this specialty.

As regards the content of teaching, all schools are delivering teaching on dementia and cognitive assessment and the majority (91%) are teaching on delirium. It is concerning that not all schools cover affective and psychotic disorders (82% and 77%, respectively) in this age group as, unlike dementia and delirium, these topics are less likely to be covered by other specialties, and their presentation and management differ considerably compared with general adult psychiatry. As with the original survey,^7^ cultural issues were covered less (50%). Academic involvement in teaching did not lead to any significant difference in the content of teaching, which should be expected if medical schools are using a standard curriculum and is perhaps reassuring given that not all medical schools will have academic old age psychiatrists involved in the organisation of teaching.

In contrast to the original survey, where student assessment was most commonly by a ‘formal examination’, this survey showed that an OSCE was the most commonly used method of assessment, with other techniques, such as assessed coursework and portfolios or logbooks, less frequently reported (Table 3). The need to ensure that teaching and assessment in medical schools is done to a high standard is crucial as assessment drives learning. However, we do not advocate a standardised assessment process as there are contextual variations in teaching nationally. What is important for educators is to understand what is being assessed and thereby select the correct assessment format as per the Millers Pyramid,^12^ i.e. multiple choice questions to test knowledge, OSCEs to assess performance. This would enable assessment processes to be undertaken in an appropriate manner and for schools to demonstrate that learning outcomes have been achieved by students.

### Implications for recruitment

Given the concerns about recruitment into old age psychiatry, it is important to consider the influence of the undergraduate curriculum experience of old age psychiatry on postgraduate career choice. A survey of graduates from Liverpool University 5 years post-qualification^13^ found that the majority felt their career choice was primarily dictated by their postgraduate experience rather than their undergraduate clinical attachments. However, in another survey of graduates, Goldacre *et al*^14,15^ found that factors during undergraduate experience significantly outweighed any inclinations before entry to medical school concerning the influence on career choice. They also found that career choices were greatly influenced by a particular teacher or department. The importance of a good role model has also been identified by surveys in other specialties,^16–18^ highlighting the importance of individuals in fostering enthusiasm and interest in a specialty.

Specialties with similar recruitment problems to old age psychiatry have found that the most significant factors influencing final-year medical students in their career choice were clinical mentors and specialty-themed, problem-based learning cases.^18^ However, for psychiatry as a whole, other studies have found that attitude changes towards considering a career in this specialty were similar whether students were taught with problem-based learning or with a more traditional curriculum.^19,20^

In elderly care medicine, a study done at the University of Aberdeen^21^ found that an intensive 8-day programme increased the likelihood of fourth-year medical students considering this specialty as a career. Several US studies have shown that a positive attitude towards older people increases the likelihood of pursuing a career in care of the elderly and that increased exposure to this specialty during medical school has a positive influence on attitudes.^22–24^ This supports the view that the most effective interventions to increase recruitment of elderly care physicians should focus on positively influencing medical students' attitudes during medical school through meaningful experiences during clinical attachments, findings which could be extrapolated to include old age psychiatry. Indeed, US and Canadian surveys looking at factors that influence medical students choosing old age psychiatry as a career have found that one of the key factors is completing an old age psychiatry rotation alongside specific teacher attributes and training experiences.^25,26^ These findings support our key recommendations (Box 1).

### Strengths and limitations

The response rate to this survey was good (74%), although it must be acknowledged that there may still have been a response bias, with a poorer return from the schools without a strong academic old age psychiatry department or representative for undergraduate teaching provision in old age psychiatry. Consequently, we may have underestimated the poorest end of the spectrum.

**Box 1** Key recommendations

1. Old age psychiatry should be offered as a clinical attachment in all medical schools.
2. All schools should deliver specific old age psychiatry outcomes in the undergraduate curriculum including affective and psychotic disorders, legal and cultural issues.
3. A minimum time delivering formal teaching and time spent on clinical attachment should be agreed by the College with medical schools to ensure adequate exposure in old age psychiatry.
4. Schools should strive to drive up the number of old age psychiatrists with teaching roles and ensure they are supported in delivering a greater diversity of teaching methods and acting as positive role models.
5. There should be greater use of patients and carers in teaching to help foster positive attitudes.
6. The use of interprofessional learning should be recognised in driving up the status of old age psychiatry and ability to practise effectively in collaboration with other professions.
7. There is a need to ensure that schools teach and assess to a similar high standard in order to demonstrate that old age psychiatry outcomes have been addressed.
8. Future surveys should look at surveying medical students on their opinion and experience of old age psychiatry as well as career intentions.

Of the responding schools, data were collected from only one representative, with the hope that the respondent identified from each school would be whoever had suitable knowledge of the undergraduate curriculum. There is a possibility, however, that some of these representatives may have had an inadequate or biased overview of the undergraduate curriculum and that not all information was reported or accurate.

It was apparent through some of the free-text responses that some schools found it difficult to quantify the length of time spent teaching curriculum outcomes specific to old age psychiatry. For medical schools with an integrated curriculum, it may have been difficult to extract this information, as there is an overlap with other specialties who may have delivered this teaching.

It must be acknowledged that a limitation in the comparisons of academic old age psychiatrist involvement is that results reported were uncorrected and it is likely that there may have been no significant difference due to the overall numbers being low, and the number of comparisons being made. A further limitation was that the survey explores the taught curriculum and does not cover student-selected topics or the fact that some medical students may choose to do a research period in old age psychiatry. There are also no data regarding the opinion of medical students, and subsequently the influence of individual schools' undergraduate experience on career intention. Consequently, the assumption could not be made that medical schools with a mandatory clinical attachment in old age psychiatry and embracing more innovative methods of teaching delivery had a positive effect on intention to pursue old age psychiatry as a career. Further research is needed to explore the experience of the curriculum on paper and the ‘hidden curriculum’ experienced by the students by means of surveys and focus groups of undergraduate students as well as following up cohorts of students into their chosen career.

### Recommendations

With the ageing population and increasing complexity of their needs, it is imperative that the future generations of doctors are suitably equipped with the knowledge, skills and attitudes for dealing with future challenges. It is especially important that the undergraduate experience fosters positive attitudes about old age psychiatry as a specialty and potential future career to try to address the workforce crisis we currently face. This survey of teacher practice has highlighted that there is still variation across medical schools in how old age psychiatry is taught, and made recommendations in how undergraduate experience of old age psychiatry can be enhanced. The value of interprofessional learning, as well as more involvement of patients and carers, should be recognised in improving the attractiveness and status of old age psychiatry. Although there may have been progress over the past 15 years in embedding old age-specific outcomes in the undergraduate curriculum, the consensus of opinion does continue to be that more curriculum space needs to be given to old age psychiatry.

## Appendix Survey regarding old age psychiatry teaching in undergraduate medical schools

1. Which medical school are you responding on behalf of? ————
2. What is your job title? ———
3. What is the current staffing establishment in academic old age psychiatry?
  (number of posts 0 1 2 3 4 5 >5) (professor/reader/senior lecturer/lecturer/research fellow/research assistant)
4. Which of the following best describes the style of teaching at your medical school? (traditional (i.e. lectures and tutorials during years 2–3 followed by the clinical years), integrated (i.e. clinical attachments from year 1), problem-based (i.e. student-centred teaching), other.)
5. Does the undergraduate curriculum contain specific old age psychiatry outcomes? (yes/no/don't know)
6. Which topics are covered? Tick all that apply (cognitive assessment/dementia/delirium/affective disorders/psychotic disorders/depression/service organisation/Mental Health Act/cultural issues/other/none of the above)
7. How much time is spent teaching old age psychiatry in the undergraduate curriculum? (e.g. approximate number of days) ————
8. When is dementia and cognitive assessment first covered? (first year/second year/third year/fourth year/fifth year)
9. Are there plans to incorporate the recent Health Education England dementia curriculum into the teaching program? (yes/no/don't know)
10. Who delivers the majority of the teaching on cognitive assessment and dementia? (old age psychiatrist/other (please specify))
11. Is there a clinical attachment in old age psychiatry? (yes/no)
12. Is the duration of a clinical attachment in old age psychiatry dependent on individual student choice? (yes/no/compulsory attachment with opportunity to spend more time on old age psychiatry/compulsory attachment with no further opportunity to spend more time on old age psychiatry)
13. Which teaching methods are used for old age psychiatry teaching? Tick all that apply (lectures/tutorials/bedside teaching/problem-based learning/inter-professional/e-learning/simulation/home visits/joint teaching/other)
14. Are carers or patients involved in the delivery of teaching? (yes/no)
15. Which methods are used to examine students on old age psychiatry? (formal examination/OSCE/long case/MCQ/coursework/e-portfolio/logbook/essay/other)
16. Are academic old age psychiatrists involved in the organisation and delivery of undergraduate teaching of old age psychiatry? (yes/no/don't know)
17. Are academic old age psychiatrists represented on your board of studies or equivalent? (yes/no/don't know)
18. Do you think that there is sufficient curriculum space given to old age psychiatry? (yes/no)
19. Any other comments re: teaching of old age psychiatry? ———
20. Would you like a copy of the results? (yes/no)
